# Effect of Sm and Ni co-doping on optical and electrical properties of lead magnesate niobate titanate ceramics

**DOI:** 10.1371/journal.pone.0357271

**Published:** 2026-09-01

**Authors:** Denial Aias, Vasilii Balanov, Neamul H. Khansur, Viktorija Pankratova, Sivagnana Sundaram Anandakrishnan, Suhas Yadav, Stanislav Cichoň, Wei Cao, Ilya Grinberg, Yang Bai

**Affiliations:** 1 Advanced Electronics Centre, University of Oulu, Oulu, Finland; 2 Department of Chemistry, Bar-Ilan University, Ramat Gan, Israel; 3 Institute of Glass and Ceramic, Friedrich-Alexander-Universität Erlangen-Nürnberg (FAU), Erlangen, Germany; 4 Department of Materials Science and Engineering, Case Western Reserve University, Cleveland, Ohio, United States of America; 5 Nano and Molecular Systems Research Unit, University of Oulu, Oulu, Finland; 6 Institute of Physics of the Czech Academy of Sciences, Prague, Czechia; National Research Centre, EGYPT

## Abstract

The ability to manipulate the bandgap while maintaining strong ferroelectricity in perovskite oxides offers numerous functional advantages, including multimodal energy harvesting/sensing, current modulation, dual-source actuation, electric-free poling, and enhanced bulk photovoltaic effect. However, bandgap engineering of Pb(Mg_1/3_Nb_2/3_)O_3_-PbTiO_3_ (PMN-PT) – renowned for its outstanding piezoelectric properties – via Ni^2+^ doping remains unexplored, despite its prior success in alkaline niobates. This study provides a comprehensive characterization of the structure-property relationships in Sm/Ni co-doped PMN-PT ceramics, especially for their optoelectrical properties. The results show that Ni^2+^ doping behaves differently than in alkaline niobates. Nevertheless, Sm/Ni co-doping effectively tunes the phase transition, photoconductivity, charge carrier trapping, and optical absorption while retaining reasonable piezoelectric energy harvesting performance. Overall, this work offers insights into the role of Ni^2+^ in the band structure of Sm-PMN-PT perovskite and pyrochlore phases.

## Introduction

In the early 2010s, Ni^2+^ doping was proposed as a strategy to engineer the bandgaps of polar oxide perovskites with the ABO_3_ structure [1, 2]. In these materials, the optical bandgap is typically defined by transitions between O 2p states forming the valance band maximum (VBM) and B-site transition metal d states forming the conduction band minimum (CBM). The strong electronegativity difference in these B-O bonds produces large spontaneous polarization, making many ABO_3_ perovskites, including PbTiO_3_, BaTiO_3_, and KNbO_3_, excellent ferroelectric, piezoelectric, and pyroelectric materials for sensors, actuators, transducers, and energy harvesters.

However, the same bonding characteristics generally result in wide bandgaps exceeding 3 eV, limiting visible-light absorption. Lower bandgaps have mainly been reported in the BiMeO_3_ family, where compounds such as BiFeO_3_, Bi(Fe_0.5_Cr_0.5_)O_3_, and BiMnO_3_ achieve bandgaps of approximately 2.7 eV, 1.4 eV, and 1.2 eV, respectively, while retaining useful ferroelectricity when the appropriate crystal structures are maintained [3–5].

Grinberg *et al.* predicted by density functional theory (DFT) that substituting Nb^5+^ with Ni^2+^ and simultaneously creating oxygen vacancies could reduce the KNbO_3_ bandgap from over 3.5 eV to about 1.1 eV through the formation of Ni^2+^-oxygen-vacancy defect dipoles [1]. Subsequent experimental studies by Bai *et al.* confirmed this mechanism in KNbO_3_ and (K,Na)NbO_3_ systems, achieving improved visible-light absorption accompanied by increased photoconductivity while maintaining remanent polarizations of 3−15 µC cm^-2^ [6–8]. In these compositions, Ba^2+^ was co-doped on the A-site to compensate for the changes in tolerance factor and charge balance introduced by Ni^2+^ doping. Similar approaches have been explored in other perovskite oxides [9–11].

Bandgap-engineered ferroelectrics offer opportunities for applications leveraging the optical modulation or multimodal coupling of electrical properties [12–14]. Despite this potential, the optical and electrical behavior of Ni^2+^-doped ABO_3_ perovskites remains insufficiently understood. Notably, the role of Ni^2+^ in photoferroelectric properties has not yet been explored in Pb(Mg_1/3_Nb_2/3_)O_3_-PbTiO_3_ (PMN-PT), one of the most technologically important piezoelectric systems.

In this study, Ni^2+^ is introduced to the B-site of PMN-PT ceramics together with Sm^3+^ co-doping on the A-site. Here, Sm^3+^ is analogous to the role of Ba^2+^ in alkaline niobates introduced above. PMN-PT was selected because compositions near the morphotropic phase boundary exhibit outstanding piezoelectric properties, making them attractive candidates for future photoresponsive piezoelectric devices and multi-source energy harvesters [15,16]. Sm^3+^ doping has also been reported to enhance the piezoelectric performance [17,18]. Because the Sm/Ni co-doped PMN-PT system is too compositionally complex for practical DFT treatment, particularly given the strong correlated Ni 3d and Sm 4f electrons, experimental investigation provides the most feasible route for understanding its behavior. This work therefore presents a comprehensive study of the structure, microstructure, and optical/electrical properties of Sm/Ni co-doped PMN-PT ceramics.

## Materials and methods

### Sample preparation

Ceramic samples were produced using the solid-state method, starting with reactants of PbO (99.9%, Sigma-Aldrich, USA; 99.9%, Thermo Scientific, USA), MgO (≥ 99%, Sigma-Aldrich, USA), Nb_2_O_5_ (99.9%, Sigma-Aldrich, USA; 99.9%, Alfa Aesar, USA), TiO_2_ (99.9% Sigma-Aldrich, USA; 99.8%, Alfa Aesar, USA), Sm_2_O_3_ (99.9%, Aldrich, USA), and NiO (99.999%, Aldrich Chemistry, USA). The reactants were accurately weighed according to the stoichiometries, and the mixtures were ball milled at 150 rpm for 6 h in a 500 ml ZrO_2_ jar with ethanol and 3 mm ZrO_2_ beads. The ceramic slurries were dried in an oven at 80–120 °C overnight. Calcination was carried out in a muffle furnace at 860 °C for 4 h, with the mixed reactants placed in an Al_2_O_3_ crucible. The calcined powder was ball-milled at 150 rpm for 12 h.

A 5 wt% binder (5 w/v% polyvinyl alcohol dissolved in deionized water) was employed to facilitate the shaping of green bodies from the calcined powders. Green body pellets, each with a diameter of 10 mm, were uniaxially pressed under a pressure of approximately 40 MPa. The green bodies were then fired at 550 °C for 4 h with a heating rate of 2 °C per minute to eliminate the binder, followed by sintering at 1200–1250 °C for 4 h in a muffle furnace. Powder beds of identical compositions were utilized to prevent Pb loss during the high-temperature treatment. After sintering, some samples were annealed in an N_2_ atmosphere at 1000 °C for 2 h.

For this study, a total of six types of specimens were prepared, as outlined in Table 1. As indicated in Table 1, the A sample family was designed to be a 71PMN-29PT, doped with 2.5 mol% Sm on the A-site. After sintering at 1250 °C in the air for 4 h, a predominant perovskite phase, referred to as A_per, was formed. By further annealing A_per in a N_2_ atmosphere at 1000 °C for 2 h, a pyrochlore phase was formed, which replaced the original perovskite phase as the major phase, and was designated as A_pyr. The B and C sample families were designed with an additional 5 mol% Ni doping into the A samples.

**Table 1:** Summary of specimens in this work.

Sample ID	Designed composition	Sintering conditions	Annealing conditions	Major phase	Normalized composition according to EPMA (electron-probe microanalysis) results	Density (g cm^-3^)
**A_per**	2.5 mol% Sm-doped 71PMN-29PT	1250 °C in air, 4 h	–	Tetragonal perovskite *P*4*mm*	(Pb_1.04 ± 0.01_Sm_0.04±0.005_)(Mg_0.21 ± 0.01_Nb_0.48±0.01_Ti_0.31 ± 0.01_)O_3.12 ± 0.02_	7.65 ± 0.02
**B_per**	2.5 mol% Sm and 5 mol% Ni co-doped 71PMN-29PT; Ni^2 +^ occupying the Mg^2 +^ -sites	1200 °C in air, 4 h	–	Tetragonal perovskite *P*4*mm*	(Pb_1.05 ± 0.02_Sm_0.04±0.005_)(Mg_0.16 ± 0.01_Ni_0.05±0.005_Nb_0.47 ± 0.01_Ti_0.33±0.01_)O_3.13 ± 0.02_	7.82 ± 0.01
**C_per**	2.5 mol% Sm and 5 mol% Ni co-doped 71PMN-29PT; Ni^2 +^ occupying the Nb^5 +^ -sites	1200 °C in air, 4 h	–	Tetragonal perovskite *P*4*mm*	(Pb_1.04 ± 0.01_Sm_0.05±0.005_)(Mg_0.18 ± 0.005_Ni_0.04±0.005_Nb_0.45 ± 0.04_Ti_0.34±0.01_)O_3.12 ± 0.02_	7.65 ± 0.03
**A_pyr**	2.5 mol% Sm-doped 71PMN-29PT	1250 °C in air, 4 h	1000 °C in N_2_, 2 h	Cubic pyrochlore *Fd*-3*m*:2	(Pb_0.76 ± 0.09_Sm_0.11±0.05_)(Mg_0.12 ± 0.05_Nb_0.59±0.07_Ti_0.29 ± 0.02_)O_3.10 ± 0.24_	–
**B_pyr**	2.5 mol% Sm and 5 mol% Ni co-doped 71PMN-29PT; Ni^2 +^ occupying the Mg^2 +^ -sites	1200 °C in air, 4 h	1000 °C in N_2_, 2 h	Cubic pyrochlore *Fd*-3*m*:2	(Pb_0.85 ± 0.05_Sm_0.14±0.03_)(Mg_0.07 ± 0.01_Ni_0.02±0.005_Nb_0.64 ± 0.04_Ti_0.28±0.04_)O_3.29 ± 0.12_	–
**C_pyr**	2.5 mol% Sm and 5 mol% Ni co-doped 71PMN-29PT; Ni^2 +^ occupying the Nb^5 +^ -sites	1200 °C in air, 4 h	1000 °C in N_2_, 2 h	Cubic pyrochlore *Fd*-3*m*:2	(Pb_0.70 ± 0.09_Sm_0.06±0.01_)(Mg_0.15 ± 0.05_Ni_0.03±0.01_Nb_0.50 ± 0.05_Ti_0.33±0.02_)O_2.87 ± 0.18_	–

However, it is important to note that Pb(Ni_1/3_Nb_2/3_)O_3_-PbTiO_3_ (PNN-PT) is also a commonly researched, stable solid solution which can form PNN-PMN-PT solid solutions in conjunction with PMN-PT [19]. To differentiate the intention of creating Ni^2+^-oxygen-vacancy defect dipoles (Ni-V_O_) from the stoichiometric PNN-PT solid solution, the 5 mol% Ni dopant was introduced in distinct ways. In the B family, the 5 mol% Ni was designed to occupy the Mg-sites by correspondingly reducing the MgO concentration in the mixture of the starting reactants, thereby creating a Mg-deficient off-stoichiometry. In the C family, the 5 mol% Ni was designed to occupy the Nb-sites by correspondingly reducing the Nb_2_O_5_ in the mixture of the starting reactants, thereby creating a Nb-deficient off-stoichiometry. As a result, the B family emulated the PMN-PNN-PT ternary solid solution with a minor stoichiometric PNN component, serving as the control specimen, while the C family aimed to create Ni-V_O_, serving as the experimental specimen.

In a manner similar to the A family, the B and C samples were sintered at 1200 °C in air for 4 h, resulting in the formation of major perovskite phases, referred to as B_per and C_per in Table 1, respectively. Corresponding pyrochlore phases, namely B_pyr and C_pyr, emerged as the major phases after further annealing the B_per and C_per samples under the same conditions that were applied to the A_per samples.

### Characterization

The calcination temperatures were determined using DSC/TGA (differential scanning calorimetry/thermogravimetric analysis, STA449 F3, Netzsch, Germany). The structures of the sintered and annealed ceramic samples were identified using XRD (X-ray diffraction, Bruker D8 Advance eco, equipped with a Cu source and a position selective detector, Germany). Diffraction data were collected in the reflection geometry between the 2θ range of 15−80 ° with a step size of 0.02 ° and acquisition time of one second at each step. Rietveld refinement was carried out using the SmartLab Studio II software and the PDF-5+ for accessing the ICDD database. The samples, after being sintered and annealed, were polished using silicon carbide abrasive papers with grit sizes ranging from P1200 to P2500. This was followed by further polishing on a plate with a suspension that had a particle size of 1 µm (Struers, France). The surfaces of the polished samples were examined under a field-emission scanning electron microscope (FESEM) equipped with energy-dispersive X-ray spectroscopy (EDX) (ULTRA plus, Zeiss, Germany). Additionally, the samples were examined using electron-probe microanalysis (EPMA, JXA-8530F Plus, JEOL, Japan) and X-ray photoelectron spectroscopy (XPS, ESCALAB 250Xi, Thermo Fisher Scientific, USA) for identification of composition and stoichiometry.

The optical properties were initially characterized using UV-vis-NIR spectrophotometry (Cary 500 Scan, Varian, USA). The thickness of all the measured samples was controlled to be approximately 200 µm to ensure comparability among the collected data. The spectrophotometry was followed by luminescence spectroscopy, which was performed under synchrotron radiation excitation at 10 K in the photoluminescence (PL) end station FINESTLUMI of the FinEstBeAMS undulator beamline at the MAX IV synchrotron facility (Sweden) [20–22]. The excitation energy range was between 4.5 and 7.5 eV, and the excitation spectra were normalized using a calibration curve obtained with the AXUV-100G diode. Luminescence detection in the visible spectral range (520–800 nm) was carried out using the Andor Shamrock (SR-303i) 0.3 m spectrometer, with a grating of 300 l mm^-1^ and a blaze of 500 nm. The spectrometer was equipped with a CCD camera (Newton DU970P-BVF, Oxford Instruments, UK) that covers the 300–1100 nm spectral range.

Prior to electrical characterizations, the sample surface was coated with a silver paste (735825, Sigma-Aldrich, South Korea) and cured at 150 °C. The dielectric and ferroelectric properties at room temperature (RT) were measured using an LCR meter (E4980AL, Keysight, USA) and a ferroelectric evaluation system (RT6000HVA, Radiant Technologies, USA), respectively. The piezoelectric properties at RT were characterized by a Berlincourt meter (YE2730A, APC International, USA) and an impedance analyzer (E4990A, Keysight, USA). The temperature-dependent dielectric parameters and I-V (current-voltage) curves were obtained using another LCR meter (4284A, Hewlett Packard, USA) and a source meter (2450, Keithley, USA), respectively. A sample stage equipped with coaxial probes (THMS 600) and its corresponding controlling unit (TMS 94/LNP, Linkam, UK) were used to control the measurement temperature. Monochromatic lasers (OBIS LX/LS series, Coherent, USA) with wavelengths of 405 nm, 552 nm, and 660 nm at a nominal power of 20 mW served as the light source for the I-V curve measurement. For I-V curve measurements at RT, a triaxial probe station (PVX400, Wentworth Laboratories, UK) was employed for more precise signal processing.

For the I-V curve measurement, it is important to note that an in-plane electrode configuration was established by depositing a pair of Au electrodes on the sample surface, maintaining a gap of approximately 150 µm. This configuration is depicted in S1 Fig.

## Results and discussion

All original datasets and their corresponding analyses related to this work are openly accessible [23].

### Phases, microstructure, and compositions of perovskites

**Fig 1:**
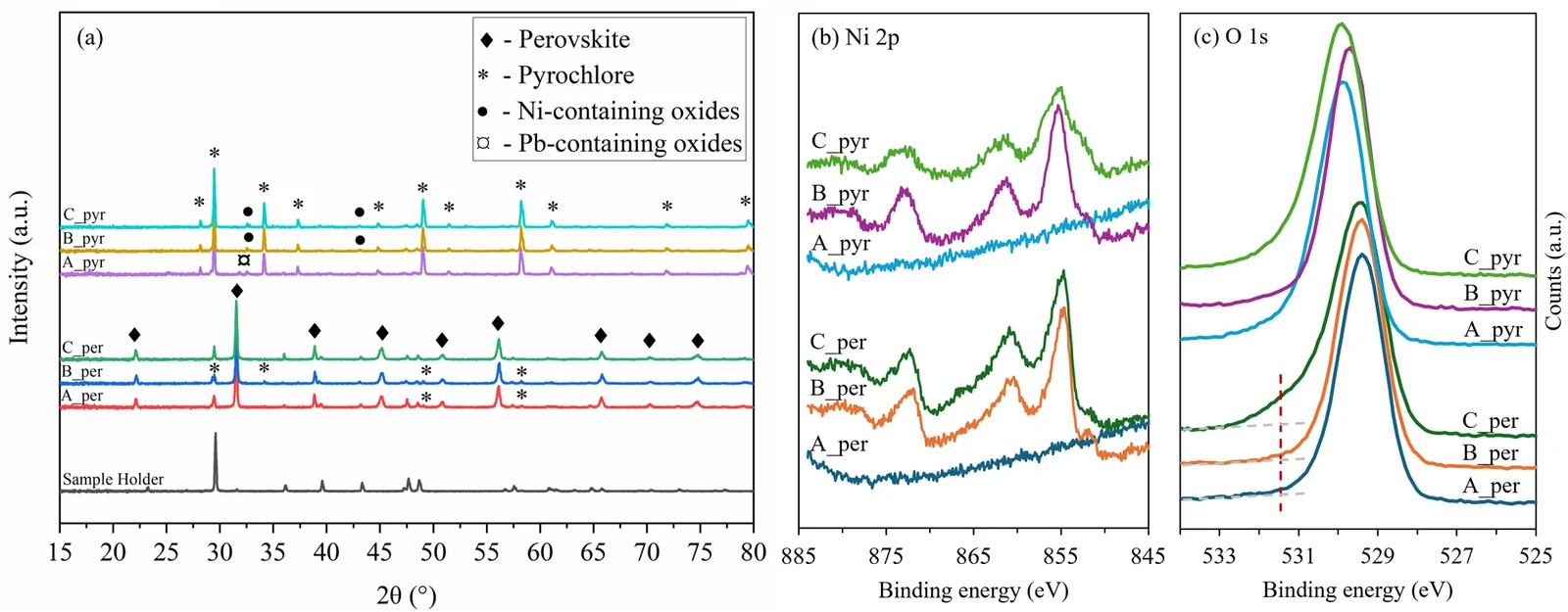
XRD and XPS results. (a) XRD patterns of the samples made in this work and the clay used to hold the samples in place during measurement; XPS results of (b) Ni 2p and (c) O 1s for the samples.

Fig 1a presents the results of the XRD analysis. The Rietveld refinement results are shown in S2 Fig., which confirmed a reasonably good fitting between the measured and calculated patterns. The refinement/instrument parameters, peak lists and other detailed refinement data can be found in the associated datasets [23]. S3–S9 Figs. depict the FESEM/EDX and EPMA images in which the perovskite and pyrochlore phases can be located. According to the XRD patterns, predominant tetragonal perovskite phases have been identified in the A_per, B_per, and C_per samples, which yielded more than 95% relative density (Table 1). The pristine A_per sample and the Ni-doped B_per sample also exhibited minor pyrochlore phases, a common occurrence in Pb-based perovskite oxides. The existence of pyrochlore phases in the A_per and B_per samples was further corroborated by the EDX and EPMA maps (S3–S4 and S9 Figs.). Although the Rietveld refinement also suggested a minor pyrochlore phase in the C_per sample, this phase was not discernible in either the EDX or EPMA maps (S5 and S9 Figs). This suggests that the pyrochlore phase could be disregarded in the C_per sample.

Upon examining the EPMA results (Table 1), it is evident that the B_per and C_per samples indeed generated distinct off-stoichiometries among Mg, Ni, and Nb. The B_per sample produced a Mg/Nb ratio of 1/2.9, whereas this ratio for the C_per sample stood at 1/2.5. By adjusting the ratios through the integration of the Ni concentration into the Mg-site, the nominal (Mg + Ni)/Nb ratios were established at 1/2.2 and 1/2.0 for the B_per and C_per samples, respectively. When compared to the original Mg/Nb ratio of 1/2.3 for the A_per sample, these calculations confirm the success of the Ni-doping in terms of the intended variation of stoichiometry.

Fig 1b shows the XPS results of Ni 2p for all the samples, which confirm the Ni^2+^ state as designed [24]. Fig 1c shows the XPS results of O 1s, where the C_per sample exhibited signs of an additional oxygen local environment that is much less prominent in A_per and B_per (see the shoulder between 531–532 eV). Because C_per was designed to reduce Nb^5+^ whilst introducing Ni^2+^, the strong charge imbalance tends to create oxygen vacancies, which we believe is responsible for the additional oxygen state in C_per. In comparison, A_per was designed to be fully oxidized and in B_per the replacement of Mg^2+^ with Ni^2+^ did not cause charge imbalance, and hence they show identical oxygen states. This suggests that the designed contrast of Ni-V_O_ between C_per and A_per/B_per is successfully realized.

**Fig 2:**
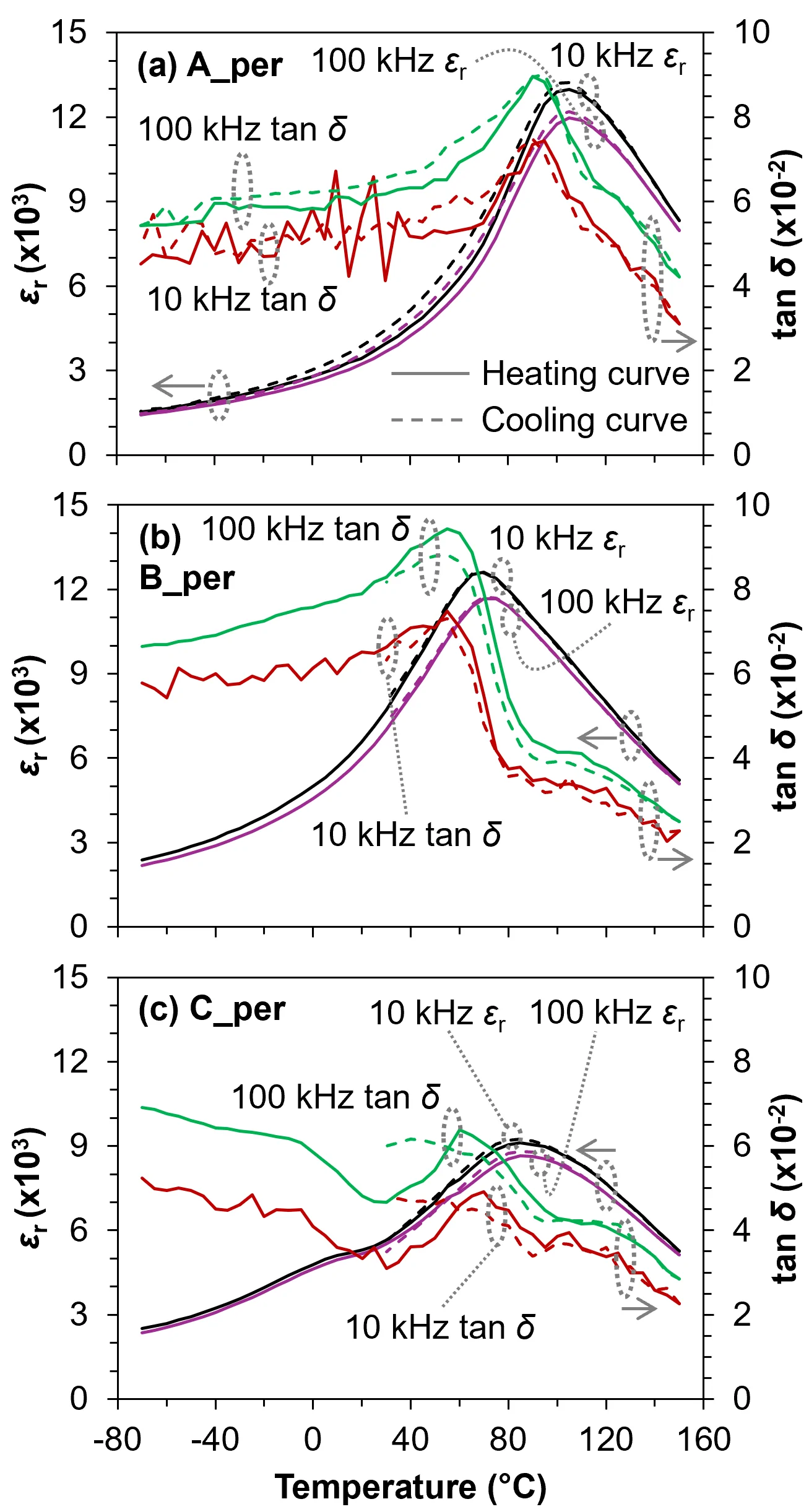
Dielectric properties. Dependence of relative permittivity (*ε*_r_) and dielectric loss (tan *δ*) on temperature measured at 10 kHz and 100 kHz under the dark condition for the A_per, B_per and C_per samples.

The distinct doping strategies also induced a significant alteration in the phase transition behavior. Fig 2 illustrates the temperature-dependent dielectric properties of the perovskite samples. The pristine A_per sample exhibited a *T*_C_ of approximately 100 °C, while the *T*_C_ of the B_per sample declined to nearly 65 °C. The C_per sample exhibited a *T*_C_ of about 85 °C. Notably, the tetragonal-cubic phase transition (around *T*_C_) in the C_per sample appeared to be considerably broader than those in the A_per and B_per samples – full width at half maximum of 100–110 °C for A_per and B_per whereas 160 °C for C_per.

The variation of *T*_C_ could be the outcome of the varying Mg/Ni/Nb ratios as discussed earlier. Grain size could also influence the *T*_C_ or phase transition behavior [25]. However, the grain sizes of the A_per, B_per and C_per samples are deemed to be similar where the negligible difference is not expected to induce as obvious changes for *T*_C_ and phase transition as those shown in Fig 2. The phase transition behavior is more likely to be altered due to the changed chemical pressure or defect concentration caused by the varying Mg/Ni/Nb ratios among the A_per, B_per and C_per samples.

### Phases, microstructure, and compositions of pyrochlores

As pyrochlore phases were not eliminated, to assist identifying possible influence of the secondary phase on the material properties, all the perovskite samples were annealed in N_2_. This process transformed all the perovskite samples into full pyrochlore phases. As depicted in Fig 1a, the samples A_pyr, B_pyr, and C_pyr exhibited dominant pyrochlore phases (space group *Fd*-3*m*:2). The precise compositions of these phases were calculated based on the EPMA results (Table 1). The FESEM images, along with the EDX and EPMA maps, revealed a significantly porous microstructure and showed no evidence of the retained perovskite phase (S6–S9 Figs). The XPS results suggest the Ni^2+^ state in A_pyr, B_pyr and C_pyr are identical to the state before annealing. An additional oxygen local environment, which is more prominent in A_pyr and C_pyr than in B_pyr, is also observed in the pyrochlore samples.

### Optical and optoelectrical behavior of perovskites

**Fig 3:**
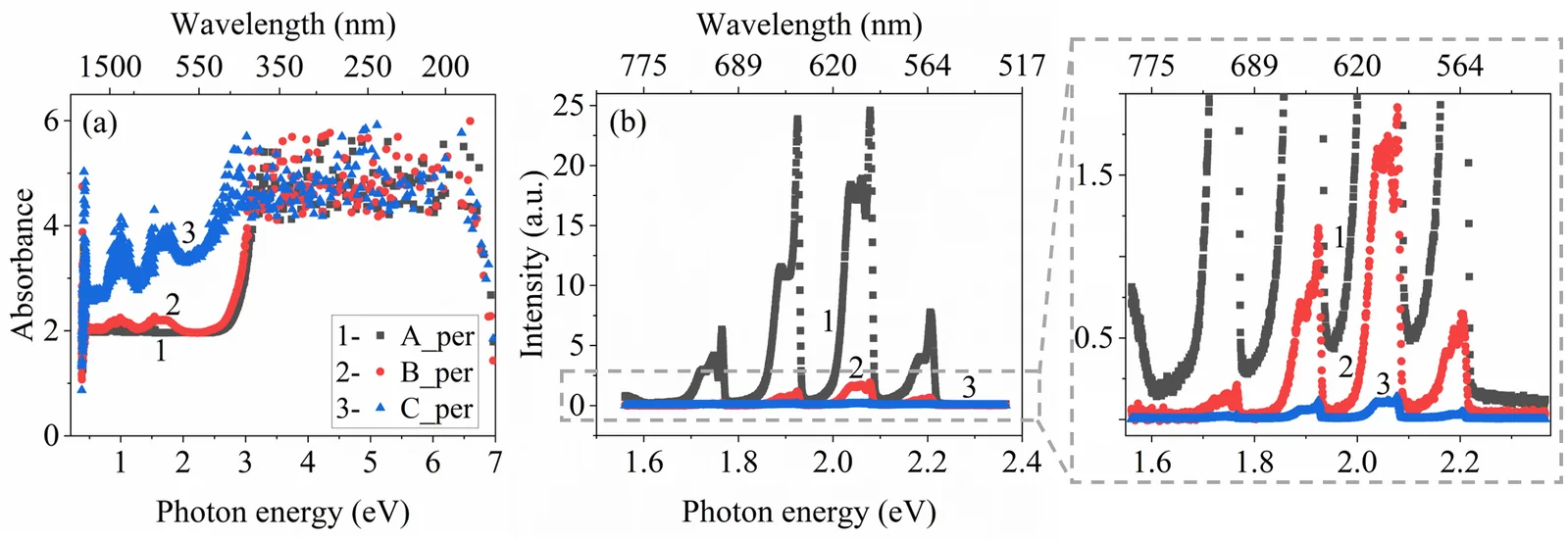
Optical properties. Dependence of (a) absorbance and (b) PL intensity on incident photon energy/wavelength for the A_per, B_per, and C_per samples.

Fig 3 presents the UV-Vis-NIR spectrophotometry results for absorbance and the PL spectroscopy results with a photon excitation energy of 40 eV for the perovskite samples. The transmittance and Tauc plots are depicted in S10 Fig, while the luminescence excitation-emission map and integral excitation signal can be found in S11 Fig. Fig 3a distinctly illustrates a manipulated absorption edge at approximately 2.2 eV for the C_per sample, in contrast to the ~ 2.4 eV for the B_per and ~2.6 eV for the A_per samples. Additional absorption peaks are also visible at around 1 eV and 1.6 eV, with the C_per sample demonstrating the strongest response.

The observed absorption peaks below the above-mentioned absorption edges, for example, at approximately 1 eV and 1.6 eV, cannot be attributed to the Sm^3+^ ions or secondary phases. A_per, B_per and C_per samples contain the same concentration of the Sm^3+^ dopant. If Sm^3+^ played a major role here, the three samples should have shown similar absorption strengths but in fact, the peak absorbance at 1 eV and 1.6 eV decreased from C_per to B_per by an order of magnitude and then from B_per to A_per by another order of magnitude, significantly contradicting the expectation. Similarly, C_per contains the least concentration of the pyrochlore phase and should have shown the weakest absorption among the three samples, if the pyrochlore phase was decisive; however, in contrast, C_per showed the strongest absorption at 1 eV and 1.6 eV. The samples also contain another secondary phase, MgO (in A_per) or (Mg,Ni)O (in B_per and C_per) that could not be detected by XRD but is visible under EDX (S3–S5 Figs). However, both MgO and NiO are known to have bandgaps much wider than 1.6 eV, and hence such a secondary phase is not an influencer in Fig 3 either.

Instead, the difference in optical properties is very likely dominated by the Ni^2+^ dopant as well as the perturbation it caused in the local chemical and defect environment inside the samples. The first evidence is that transmission and the direct-gap and indirect-gap Tauc fitting generally align with the absorption results (S10 Fig). Similar effects have been reported following the doping of Ni^2+^ into Na_0.5_Bi_0.5_TiO_3_-BaTiO_3_ and KNN-(Bi_0.5_Na_0.5_)ZrO_3_ solid solutions [10,11].

The second and more convincing evidence is that, according to Fig 3b, the PL spectra displayed several peaks with maximum emissions at 562, 597, 645, and 703 nm. The signal shapes are consistent across all the samples measured. The emission spectra showed sharp lines that are consistent with the expected Sm^3+^ emission, for example, the ^4^*G*_5/2_ → ^6^*H*_*ι*_ (*ι* = 5/2, 7/2, 9/2, 11/2) transitions [26,27]. However, a stark contrast is observed in the signal intensity among different samples. The intensity diminished by an order of magnitude transitioning from the A_per to B_per sample, and it further decreased by another order of magnitude from the B_per to C_per sample. This means the emission behavior shows exactly the opposite trend to that observed in the absorption behavior, strongly pointing towards formation of local charge traps induced by Ni^2+^ inside the bandgap.

As indicated in S11 Fig, when the photon energy exceeded 5 eV, the luminescence excitation intensity began to decrease due to the non-radiative recombination of electrons and holes. Upon reaching an excitation energy of 6.8 eV, the luminescence excitation intensity started to increase again, marking the onset of the multiplication of the electronic excitation (MEE) process. The MEE process is characterized by the formation of two or more emission centers as a result of inelastic electron-electron scattering.

The third evidence of Ni^2+^ doping playing a decisive role appears with the expanding Urbach tail. In order to achieve enhanced luminescence excitation intensity under the MEE process, the excitation energy must exceed the threshold energy, which is approximately 2–2.5 times the bandgap. Consequently, the bandgap of the A_per sample can be indirectly estimated to fall within the range of 2.7–3.4 eV. Based on the PL results, the B_per and C_per samples are expected to have a bandgap similar to that of the A_per sample. However, the absorption spectra (Fig 3a) show clear evidence of evolving Urbach tails, with the absorption edge decreasing from 2.6 eV for A_per to 2.4 eV for B_per and then further to 2.2 eV for C_per. This indicates that the Ni^2+^ doping by reducing Nb^5+^ induced strong charge imbalance and thus substantial local chemical and microstructural heterogeneity in C_per [28].

For such complex samples with microstructural inhomogeneity, the apparent absorption or luminescence behavior could not accurately represent the electrical transport gaps [29,30]. Therefore, photoconductivity was employed as a crucial tool to reveal the true electrical band structures in the samples.

**Fig 4:**
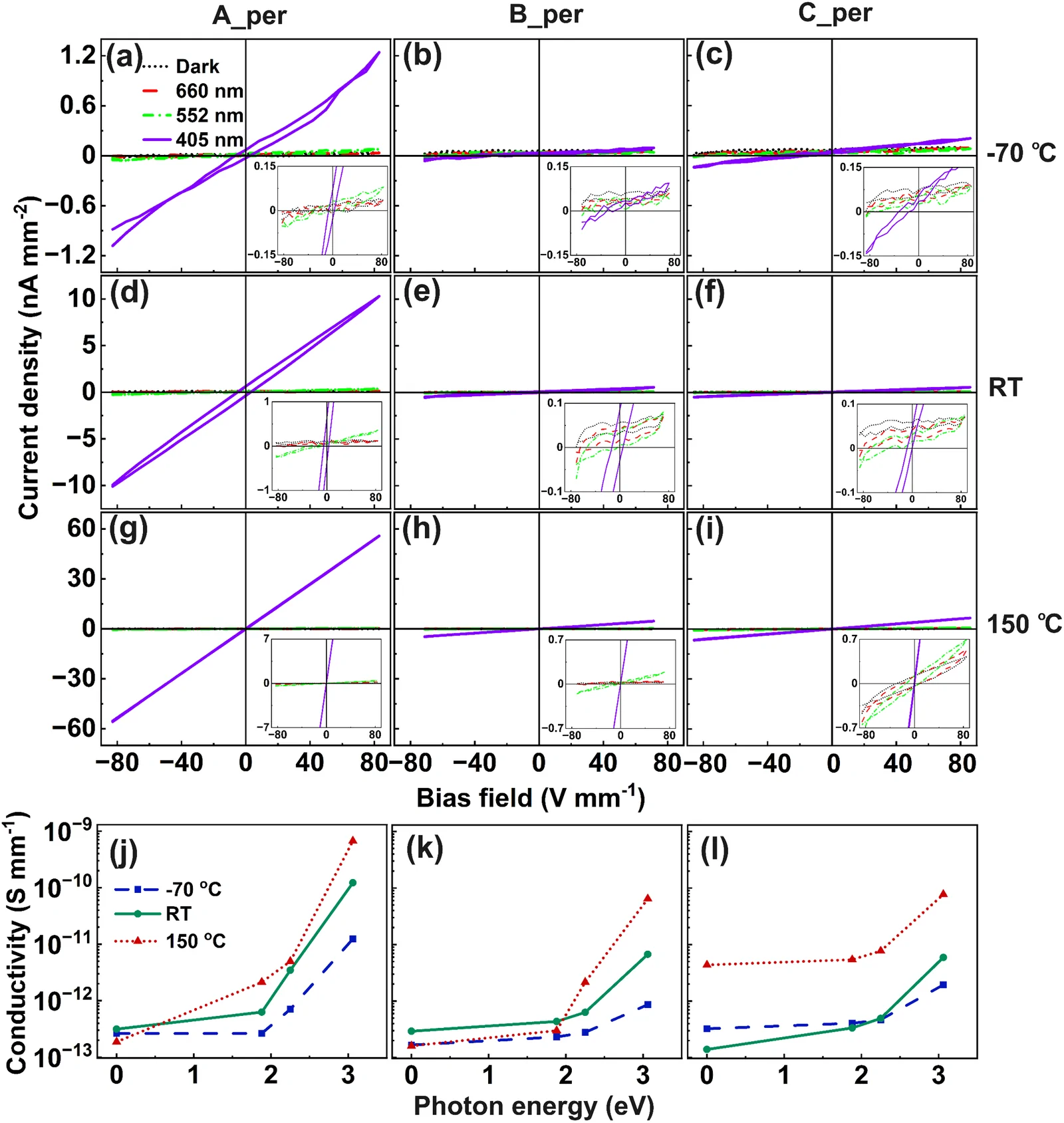
Optoelectrical properties of perovskites. (a)-(i) Dependence of current density on electric field measured at (a)-(c) −70 °C, (d)-(f) RT, and (g)-(i) 150 °C under illumination with different photon energies, and (j)-(l) dependence of conductivity on incident photon energy measured at −70 °C, RT and 150 °C, for the (a)(d)(g)(j) A_per, (b)(e)(h)(k) B_per and (c)(f)(i)(l) C_per samples.

The small-field *J*-*E* (current density-electric field) curves for the perovskite samples, measured at −70 °C, RT and 150 °C, are depicted in Fig 4, along with the calculated photoconductivity. The large-field *J*-*E* curves at RT are presented in S12 Fig. It should be noted that the terms ‘small field’ and ‘large field’ refer to the applied electric fields that are significantly smaller and larger, respectively, than the coercive fields of the samples.

The initial observation from Fig 4 is that, following the Ni doping, the B_per and C_per samples exhibited significantly lower conductivity under the 405 nm laser (3.06 eV) compared to the A_per sample under the same illumination. This phenomenon is consistent across all three measurement temperatures. The A_per sample demonstrated a distinct band-band transition under the 405 nm laser as shown in Fig 4a, d and g. Given that Fig 3 suggested a bandgap in the range of 2.7–3.4 eV, it is inferred that the actual bandgap of the A_per sample lies within the range of 2.7–3.06 eV.

However, the scenarios for the B_per and C_per samples appeared to be more complex. To facilitate the analysis, Table 2 enumerates various indicators that could suggest charge carrier transport. The absorption edge (*E*_a_) values are derived from Fig 3a. The photon energy values, which resulted in an order of magnitude higher conductivity compared to the dark value, are extracted from Fig 4j-l. As the temperature rises, the charge carriers should become increasingly mobile. However, for the A_per sample, the dark conductivity values remained similar across different temperatures (Fig 4j), with significant differences only observed in the photoconductivity values. This suggests A_per contains a clean bandgap, i.e., possible charge trapping centers between the CBM and VBM can be neglected, thereby further confirming an intrinsic bandgap of 2.7–3.06 eV, as previously discussed.

**Table 2:** Summary of possible indicators for optical and electrical transport gaps obtained from the absorption measurement, PL spectroscopy, and *J*-*E* curves.

Sample ID	Absorption edge (*E*_a_) (eV)	Bandgap suggested by PL (*E*_pl_) (eV)	Photon energy at 10 times dark conductivity (eV)		
			−70 °C (*E*_10_-70_)	RT (*E*_10_RT_)	150 °C (*E*_10_150_)
**A_per**	~2.6	2.7-3.4	~2.6	~2.2	~1.8
**B_per**	~2.4	2.7-3.4	>3.06	~2.7	~2.2
**C_per**	~2.2	2.7-3.4	>3.06	~2.6	~2.8
**A_pyr**	–	–	~2.1	~1.1	–
**B_pyr**	–	–	~1.5	~0.7	–
**C_pyr**	–	–	~2.0	~1.6	–

The B_per sample exhibited a situation analogous to the A_per sample, where the dark conductivity values at varying temperatures are comparable. It is noteworthy that for the B_per sample, the conductivity values across different temperatures also remained consistent under 1.88 eV photon energy (660 nm laser) (Fig 4k), leading to a more challenging initiation of band-band transition compared to the A_per sample. In fact, at −70 °C, the photon energy that resulted in an order of magnitude increase in conductivity (*E*_10_-70_) for the B_per sample exceeded the measurement range, 3.06 eV (Table 2). This observation implies existence of charge traps, possibly introduced by Ni^2+^, which can be further elaborated with C_per.

For the C_per sample, although the dark conductivity values at −70 °C and RT were within the same order of magnitude, the value at 150 °C jumped by an order of magnitude (Fig 4l). This cannot be simply explained by the phase transition from tetragonal to cubic, because the same phenomenon is not shown by A_per and B_per, of which the *T*_*C*_ is also below 150 °C. Instead, thermal activation of localized charge trapping centers is a more plausible cause.

Indirect corroborative evidence can be observed in S12 Fig. Upon expanding the electric field beyond the coercive field, a noticeable increase in photoconductivity under the 552 nm laser (2.25 eV) occurred in the B_per sample, but not in the C_per sample. Therefore, we attribute this discrepancy to the presence of charge centers within the bandgap of the C_per sample, resulting in the additional charges injected from the expanded electric field being captured.

The photocurrents shown in Fig 4 underwent the stages of photoexcitation and the migration of electrons under the bias voltages. Although photoexcited electrons might be created by photons from longer wavelengths (660 and 552 nm) after being excited into charge trapping centers, they were incapable to form photocurrent due to strong localization. The most significant discrepancy between the absorption measurement and the *J*-*E* curve measurement is observed in the C_per sample, where even the band conduction under photon energy of 3.06 eV was significantly suppressed. The primary evidence included the parallel evolution trends of photoconductivity at various temperatures (Fig 4l), suggesting that the photon energy triggering an order of magnitude increase in conductivity at different temperatures was nearly identical (Table 2). However, this conclusion is deemed to be skewed by the potential presence of charge traps, as will be clarified below.

### Optical and optoelectrical behavior of pyrochlores

**Fig 5:**
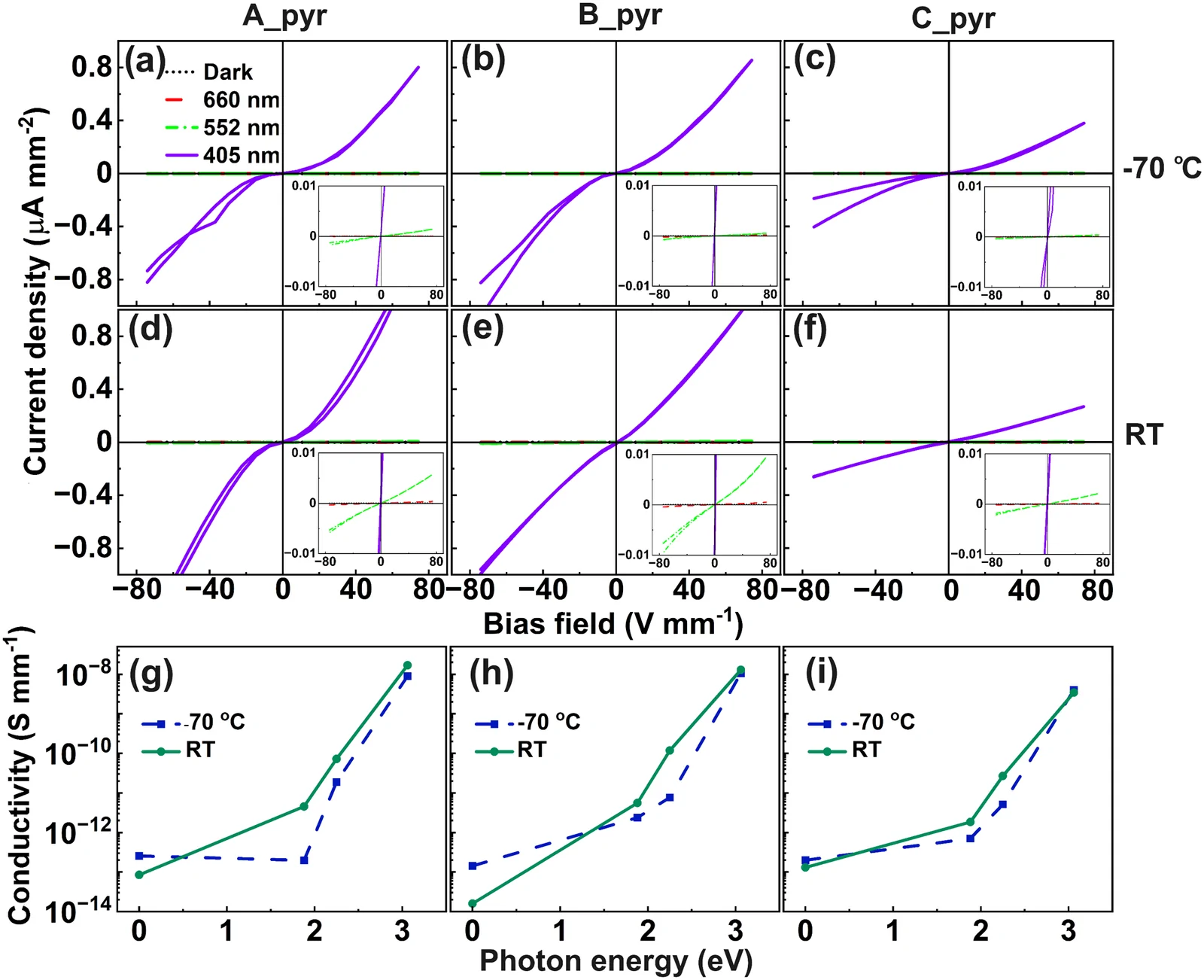
Optoelectrical properties of pyrochlores. (a)-(f) Dependence of current density on electric field measured at (a)-(c) −70 °C and (d)-(f) RT under illumination with different photon energies, and (g)-(i) dependence of conductivity on incident photon energy measured at −70 °C and RT, for the (a)(d)(g) A_pyr, (b)(e)(h) B_pyr and (c)(f)(i) C_pyr samples.

Fig 5 presents the measurement results similar to those depicted in Fig 4 but conducted for the pyrochlore samples. The pyrochlore phases were already in the cubic form at RT, and therefore, they were not tested at temperatures above RT. All the pyrochlore samples demonstrated typical narrow-gap semiconducting behavior, and the scenarios were considerably simpler compared to the perovskite samples. Electrical transport gaps that trigger drastic increase in photoconductivity can be identified, depending on the temperature, to be approximately 1.1–2.1 eV, 0.7–1.5 eV, and 1.6–2.0 eV for the A_pyr, B_pyr, and C_pyr samples, respectively (Table 2).

Interestingly, the dark conductivity values of all the pyrochlore samples are comparable to those of the perovskite samples. However, the photoconductivity values of the pyrochlore samples are generally 1–2 orders of magnitude greater than those of the perovskite samples. This suggests a potentially effective strategy in practice for adjusting optoelectrical properties by employing different components fabricated from different phases or by balancing the phase concentrations within a single component (e.g., creating perovskite-pyrochlore composites) [8,31].

The reduced transport gap values of the pyrochlore phases could also explain the weak additional absorption peaks at approximately 1 eV and 1.6 eV for A_per (Figs 1a and 3a). Given that the concentrations of the pyrochlore phases in the perovskite samples were negligible, they were unlikely to contribute to the distinct optical absorption and photoconductivity of B_per and C_per.

### Different roles of Ni^2+^-oxygen-vacancy defect dipoles in complex PMN-PT and in simple alkaline niobates

Compared with previously researched Ni-doped alkaline niobates which achieved favorable reduction in electrical transport gaps, this work shows Ni-doping is modifying the transport gaps in a dissimilar way [1,32]. After Ni^2+^ doping, PMN-PT tends to show increased structural and electronic disorder. There is unambiguous evidence: The PL intensities and photoconductivity decrease simultaneously.

First, the Sm^3+^ emission line shapes and positions remained essentially unchanged (Fig 3b-c), but there is a monotonic decrease in the PL intensity with Ni incorporation. This observation is unlikely to originate primarily from modifications of the Sm^3+^ crystal-field environment. Instead, after doping Ni^2+^ whilst reducing Mg^2+^, the newly introduced states may promote carrier localization and non-radiative recombination. This is because unlike Mg^2+^, which is a closed-shell ion and contributes little to the electronic states near the band edges, Ni^2+^ processes partially filled 3d orbitals that can introduce localized electronic states and strong electron-correlation effects [33,34]. The increased non-radiative recombination decreased the PL intensity by an order of magnitude from A_per to B_per.

This effect becomes more prominent when Ni substitutes within the electronically active Nb-O framework, i.e., after doping Ni^2+^ whilst reducing Nb^5+^. The emergence of a pronounced Urbach tail (Fig 3a), the growth of the high-binding-energy O 1s component (Fig 1c), and the broadening of the ferroelectric-paraelectric phase transition in C_per collectively indicate local disorder, implying a change towards relaxor behavior. This increased disorder further traps photoexcited charge carriers and enhances non-radiative recombination, leading to another order of magnitude decrease in PL intensity from B_per to C_per.

These localized states caused by the disorder explain the suppressed photoconductivity, as discussed above, via non-radiative carrier trapping and recombination. In comparison, in simple alkaline niobates, Ni^2+^ participates in a relatively simple Nb-O electronic network and forms defect complexes that contribute to bandgap narrowing. It should be noted that the key to success in Grinberg’s model for alkaline niobates is that a long-range Ni^2+^-oxygen vanancy-Nb^5+^ order should be established [1], whereas in the complex multi-cation B-site environment, this order can be easily disrupted, as is concluded by the experimental results in this work.

### Relationship between composition/microstructure and ferroelectric/piezoelectric properties

**Fig 6:**
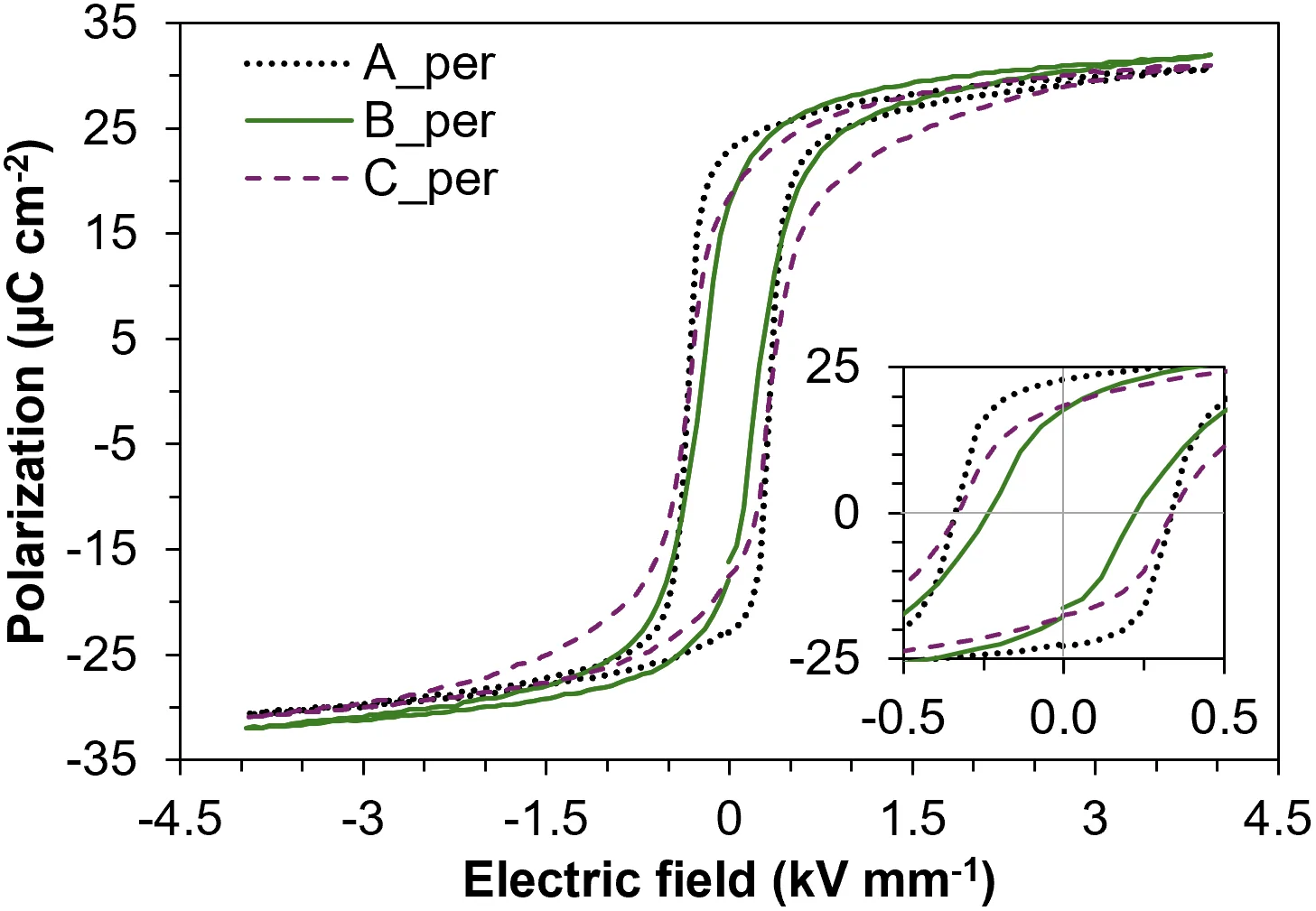
Ferroelectric properties. Dependence of polarization on electric field (*P*-*E* loops) measured at RT, at 1 Hz and under the dark condition for the A_per, B_per and C_per samples.

The pyrochlore phases are cubic at RT, thus the pyrochlore samples (A_pyr, B_pyr and C_pyr) did not exhibit any ferroelectric or piezoelectric response as expected. The existence of pyrochlore phases proves to have negligible negative impact on the remanent polarization (*P*_*r*_), as illustrated in Fig 6, i.e., the *P*-*E* (polarization-electric field) hysteresis loops of the perovskite samples, where the *P*_*r*_ value of A_per (23 µC cm^-2^) successfully replicates the results in previous reports [17]. The *P*_*r*_ values of B_per and C_per are approximately 20% lower than that of A_per.

Nevertheless, this apparently identical decrease is thought to be due to different reasons. For B_per, the decrease in *P*_*r*_ is mainly due to the measurement temperature (RT) is closer to the *T*_*C*_ of B_per. It is well established that *P*_*r*_ tends to decrease when approaching the ferroelectric-paraelectric phase transition due to increased symmetry of the crystal structure and thermal activation of domain wall motion. On the other hand, for C_per, the decrease in *P*_*r*_ is due to increased disorder and local heterogeneity, which pushes the composition towards the relaxor-behaving counterparts.

The thermal activation of domain wall motion is also evident in the coercive field (*E*_*c*_), where B_per appears to be softer than A_per and C_per. However, again, the apparently identical *E*_*c*_ of A_per and C_per is caused by different mechanisms. Treating A_per as the pristine state, Ni^2+^ doping while reducing Mg^2+^ did not create extra oxygen vacancies (Fig 1c) and thus the domain wall pinning effect did not change significantly from A_per to B_per, but the domain walls in B_per were more mobile due to the composition’s proximity to *T*_*C*_, resulting in reduced *E*_*c*_. From B_per to C_per, the composition actually became harder due to the increased concentration of oxygen vacancies (Fig 1c) that induced a larger domain wall pinning effect.

Table 3 summarizes the dielectric properties along with the pertinent piezoelectric properties. The *d*_33_ (longitudinal piezoelectric charge coefficient), *g*_33_ (longitudinal piezoelectric voltage coefficient), FOM (figure of merit for piezoelectric energy harvesting), *Q*_M_ (mechanical quality factor), and *η*_m_ (efficiency of piezoelectric energy harvesting) values were calculated in accordance with Equations 1–4 [35]. In these equations, *ε*_0_ is the permittivity in vacuum, *f*_r_ is the resonant frequency, *f*_a_ is the anti-resonant frequency, *Z*_m_ is the impedance at resonance, and *C*^T^ is the capacitance at 1 kHz after poling.

**Table 3:** Summary of dielectric and piezoelectric properties measured at RT for the A_per, B_per and C_per samples.

Sample ID	*ε*_r_ at 1 kHz (unpoled value/poled value)	tan *δ* (x10^-2^) at 1 kHz (unpoled value/poled value)	*d*_33_ (pC N^-1^)	*g*_33_ (mVm N^-1^)	FOM (x10^-12^ m^2^ N^-1^)	*Q*_M_	*η*_m_
**A_per**	4835 ± 127/ 5615 ± 231	4.52 ± 0.06/ 2.71 ± 0.07	854 ± 13	19.9 ± 0.4	17.0 ± 0.4	40 ± 2	0.93 ± 0.005
**B_per**	7822 ± 73/ 9290 ± 105	6.34 ± 0.01/ 6.13 ± 0.08	526 ± 21	7.5 ± 0.1	3.9 ± 0.1	28 ± 2	0.72 ± 0.01
**C_per**	5386 ± 303/ 6133 ± 329	3.79 ± 0.14/ 2.95 ± 0.02	590 ± 19	12.6 ± 0.5	7.5 ± 0.2	58 ± 2	0.88 ± 0.005


$$\begin{array}{c} g_{\text{3}\text{3}}=\frac{d_{\text{3}\text{3}}}{\varepsilon_{r}\cdot \varepsilon_{\text{0}}} \end{array}$$ (1)

$$\begin{array}{c} \text{FOM}=d_{\text{3}\text{3}}\cdot g_{\text{3}\text{3}} \end{array}$$ (2)

$$\begin{array}{c} Q_{M}={\left(\text{2}\pi \cdot f_{r}\cdot \left|Z_{m}\right|\cdot C^{T}\cdot \left(f_{a}^{\text{2}}-f_{r}^{\text{2}}\right)\cdot f_{a}^{-\text{2}}\right)}^{-\text{1}} \end{array}$$ (3)

$$\begin{array}{c} \eta_{m}=\frac{\left(Q_{M}+{\left(tan\;\delta \right)}^{-\text{1}}\right)\cdot \left(\frac{\left(f_{a}^{\text{2}}-f_{r}^{\text{2}}\right)\cdot f_{a}^{-\text{2}}}{\text{1}-\left(\left(f_{a}^{\text{2}}-f_{r}^{\text{2}}\right)\cdot f_{a}^{-\text{2}}\right)}\right)}{\text{2}+\left(Q_{M}+{\left(tan\;\delta \right)}^{-\text{1}}\right)\cdot \left(\frac{\left(f_{a}^{\text{2}}-f_{r}^{\text{2}}\right)\cdot f_{a}^{-\text{2}}}{\text{1}-\left(\left(f_{a}^{\text{2}}-f_{r}^{\text{2}}\right)\cdot f_{a}^{-\text{2}}\right)}\right)} \end{array}$$ (4)

From our experience, the minor pyrochlore phase, the slight difference in density of such high densification level (>95%), and the comparable grain size across the samples are not expected to influence the dielectric and piezoelectric properties in a noticeable way; the perovskite phase tends to be the sole decisive factor [36]. From Table 3, it can be observed that the permittivity of the B_per sample at RT is significantly larger than those of the A_per and C_per samples. As discussed above, this is clearly attributed to RT being closer to the *T*_C_ of the B_per sample (Fig 2). As both FOM and *g*_*33*_ are negatively related to permittivity, consequently, the FOM and *g*_33_ values of the B_per sample are nearly half of those of the C_per sample, given the similar *d*_33_ values between the B_per and C_per samples. In addition, the *η*_m_ value of the B_per sample is approximately 20% lower than that of the C_per sample, a result of the complex influence of dielectric and piezoelectric responses. FOM and *η*_m_ are indicators of the capability and efficiency, respectively, of a piezoelectric material during a kinetic energy harvesting process, while *g*_33_ signifies the sensitivity of a piezoelectric material when subjected to mechanical stimuli. Therefore, the B_per sample would be considerably less effective as an energy harvesting and sensing material compared to the C_per sample. Moreover, the B_per sample yielded a *Q*_M_ value that is more than 50% lower than that of the C_per sample, suggesting that the B_per sample would also be a far less efficient transducing material than the C_per sample.

The more favorable piezoelectric properties of C_per than B_per prove a positive consequence of the compositional and microstructural change towards relaxors. The observation also implies a possible contradiction between bandgap engineering and optimization of piezoelectric properties for multimodal energy harvesting/sensing applications. This is a less advantageous feature compared with Ni-doped alkaline niobates where minimization of electrical transport gap and optimization of piezoelectric properties can be achieved simultaneously [8].

In contrast, the A_per sample is anticipated to outperform the C_per sample in terms of actuation (with a *d*_33_ value approximately 50% higher), sensing (with a *g*_33_ value roughly 60% higher), and off-resonance energy harvesting (with an FOM value about 125% higher). The FOM value of the A_per sample is comparable with the highest value reported in the literature [17]. On the other hand, the C_per samples could surpass the A_per sample in terms of transducing, with a *Q*_M_ value approximately 40% higher. The *η*_m_ values of the A_per and C_per samples are deemed to be very similar, suggesting that these two materials would exhibit comparable energy harvesting efficiencies at resonance.

Surprisingly, the sample without Ni-doping actually showed the best overall ferroelectric, piezoelectric, and optoelectrical properties. Although A_per is thought to have a clean bandgap of roughly 3 eV, the narrow-gap, semiconducting pyrochlore phase in the microstructure likely contributed to the enhanced photoconductivity under illumination with sub-gap photon energies as a result of the minimum non-radiative charge trapping and recombination compared with the situation for B_per and C_per.

## Conclusions

This work establishes that the behavior of Ni^2+^ doping in Sm-doped PMN-PT differs fundamentally from that reported for alkaline niobate ferroelectrics. Although Ni^2+^ incorporation has previously been proposed as a route for bandgap reduction through the formation of Ni-related defect complexes, no comparable bandgap narrowing was observed in the Sm-doped PMN-PT system. Instead, the results demonstrate that the incorporation of Ni^2+^ modifies the local structural and electronic environment, leading to significant changes in dielectric, ferroelectric, optical, and optoelectrical responses.

The experimental findings further reveal that the effects of Ni^2+^ are highly dependent on the substitution strategy. Replacing Mg^2+^ and Nb^5+^ with Ni^2+^ produces distinct impacts on phase stability, ferroelectric behavior, photoconductivity, and charge carrier dynamics, indicating that the role of Ni^2+^ is governed also by its interaction with the B-site cation framework. In particular, evidence from optical absorption, photoconductivity, photoluminescence, and XPS analyses suggests that Nb-site substitute introduces a greater degree of electronic disorder and localized states, which strongly influence carrier transport and recombination processes.

Overall, via experimental works that cannot be replaced by DFT calculations due to the complex composition and strong hybridization between elements, the present results demonstrate that Ni^2+^ doping should not be regarded as a universal bandgap-engineering strategy for ferroelectric perovskites, as it is currently only proven effective on simple alkaline niobates. Nevertheless, it remains a powerful means of tuning optical absorption, photoconductivity, carrier trapping behavior, and ferroelectric phase transition characteristics in Sm-doped PMN-PT. These findings provide new insight into the interactions between dopant chemistry, electronic disorder, and optoelectrical functionality in complex relaxor ferroelectrics, and offer guidance for the future design of multifunctional piezoelectric and photoresponsive ceramic materials.

## Supporting information

S1 Fig An optical microscopy image illustrating the in-plane electrode configuration used for I-V curve measurements. The image was taken from sample A_per.
(TIF)

S2 Fig Rietveld refinement results. (a) A_per, (b) A_pyr, (c) B_per, (d) B_pyr, (e) C_per, and (f) C_pyr samples.
(TIF)

S3 Fig FESEM micrograph and EDX maps of sample A_per. (TIF)

S4 Fig FESEM micrograph and EDX maps of sample B_per. (TIF)

S5 Fig FESEM micrograph and EDX maps of sample C_per. (TIF)

S6 Fig FESEM micrograph and EDX maps of sample A_pyr. (TIF)

S7 Fig FESEM micrograph and EDX maps of sample B_pyr. (TIF)

S8 Fig FESEM micrograph and EDX maps of sample C_pyr. (TIF)

S9 Fig Micrographs taken during EPMA (electron-probe microanalysis). The number annotations are associated with the measurement data [23].
(TIF)

S10 Fig Supplementary optical properties. Dependence of (a) transmittance, (b) (F(R)·hν)^2^, and (c) (F(R)·hν)^0.5^ on incident photon energy obtained from the spectrophotometry. R is measured reflectance, h is Planck’s constant, and ν is frequency of the incident light. hν represents the incident photon energy. Theoretically, F(R) = (1-R)^2^·(2R)^-1^, which is widely used in the Tauc fitting method.
(TIF)

S11 Fig Supplementary photoluminescence results. (a) Luminescence excitation-emission map of the A_per sample; Integral excitation signal of (b) the A_per sample and (c) the C_per sample.
(TIF)

S12 Fig Supplementary optoelectrical properties. Dependence of current density on electric field measured at RT under different incident photon energy for the (a) A_per, (b) B_per and (c) C_per samples. Note here the maximum applied electric field exceeds the coercive fields of all the samples.
(TIF)
