# Supplementary material for: Effect of Sm and Ni co-doping on optical and electrical properties of lead magnesate niobate titanate ceramics
Source: PLoS One. 2026 Sep 1;21(9):e0357271. doi: 10.1371/journal.pone.0357271 (PMC13533389; doi:10.1371/journal.pone.0357271)
Supplement: Supplementary file 1 [file pone.0357271.s013.docx]

Recommendation: Major Revision

The manuscript presents a comprehensive experimental study that is methodologically rigorous and well documented. Overall, the experimental data support the conclusions, which are presented in a balanced and cautious manner. However, several aspects should be improved before the manuscript can be considered for publication in PLOS ONE. In particular, the discussion of the underlying physical mechanisms should be strengthened, certain interpretations related to the bandgap and defect chemistry require further clarification, and the results should be better discussed in the context of recent literature. After addressing the following comments, the manuscript would meet the publication standards of the journal.

Major Comments

1) The authors should provide a more detailed explanation of why Ni exhibits a different behavior in the PMN-PT system compared with alkaline niobates. The discussion should clarify the possible physical and chemical mechanisms responsible for this discrepancy and relate the observations to previous studies.
2) The oxidation state of Ni should ideally be confirmed by X-ray Photoelectron Spectroscopy (XPS). If such measurements are not available, the authors should clearly acknowledge this limitation and discuss how it may affect the interpretation of the results.
3) The discussion regarding the relationship between the microstructure and the electrical properties should be significantly improved. A deeper analysis linking the observed microstructural features to the dielectric, ferroelectric, piezoelectric, and optoelectrical behaviors would considerably strengthen the manuscript.
4) The Introduction is relatively lengthy, and the Conclusions largely repeat results that have already been discussed. The Introduction should be condensed to improve readability, while the Conclusions should focus on the main findings and their scientific significance rather than restating experimental observations.

Minor Comment

The manuscript would benefit from the inclusion of several recent and relevant references published between 2024 and 2026, particularly in the fields of photoferroelectric materials, bandgap engineering, and optoelectronic ferroelectrics.
