# Supplementary material for: Effect of Sm and Ni co-doping on optical and electrical properties of lead magnesate niobate titanate ceramics
Source: PLoS One. 2026 Sep 1;21(9):e0357271. doi: 10.1371/journal.pone.0357271 (PMC13533389; doi:10.1371/journal.pone.0357271)
Supplement: Supplementary file 2 [file pone.0357271.s015.docx]

**Response letter to reviewers**

Dear Dr. Talaat Abdel Hamid,

Thank very much for handling our manuscript for peer review. We have improved our manuscript based on your and the reviewer’s comments. Please find our response to each of the comments as follows.

**Journal Requirements:**

When submitting your revision, we need you to address these additional requirements.

1.Please ensure that your manuscript meets PLOS ONE's style requirements, including those for file naming. The PLOS ONE style templates can be found at

https://journals.plos.org/plosone/s/file?id=wjVg/PLOSOne_formatting_sample_main_body.pdf and

https://journals.plos.org/plosone/s/file?id=ba62/PLOSOne_formatting_sample_title_authors_affiliations.pdf

**Response**: We have followed the instructions provided in the above webpages. We believe our manuscript now meets the PLOS ONE’s style requirements. Please see the highlighted changes throughout the revised manuscript.

2. Please note that funding information should not appear in any section or other areas of your manuscript. We will only publish funding information present in the Funding Statement section of the online submission form. Please remove any funding-related text from the manuscript.

**Response**: We have removed all funding-related texts from the manuscript.

3. Thank you for stating the following financial disclosure:

“(1) Funded by the European Union (ERC, UNIFY, 101039110). Views and opinions expressed are however those of the authors only and do not necessarily reflect those of the European Union or the European Research Council Executive Agency. Neither the European Union nor the granting authority can be held responsible for them; (2) Co-funded by the European Research Council (ERC) under the European Union's Horizon 2020 research and innovation programme (grant agreement No. 101002219); (3) Co-funded by the Israel Science Foundation, grant number 1479/21.”

Please state what role the funders took in the study. If the funders had no role, please state: "The funders had no role in study design, data collection and analysis, decision to publish, or preparation of the manuscript."

If this statement is not correct you must amend it as needed.

Please include this amended Role of Funder statement in your cover letter; we will change the online submission form on your behalf.

**Response**: First, we have removed all funding-related texts from the manuscript and will only state the funding in the online submission form. Also, we have stated "The funders had no role in study design, data collection and analysis, decision to publish, or preparation of the manuscript." in the cover letter.

4. Please include captions for your Supporting Information files at the end of your manuscript, and update any in-text citations to match accordingly. Please see our Supporting Information guidelines for more information: http://journals.plos.org/plosone/s/supporting-information.

**Response**: We have followed the guidelines provided in the above webpage and provided captions for the Supporting Information files at the end of the revised manuscript; please see the highlighted changes.

5. If the reviewer comments include a recommendation to cite specific previously published works, please review and evaluate these publications to determine whether they are relevant and should be cited. There is no requirement to cite these works unless the editor has indicated otherwise.

**Response**: The reviewer suggested to include extra references but did not specify. We agree with the reviewer and hence have included more related references.

**Reviewer #1:**

The manuscript presents a comprehensive experimental study that is methodologically rigorous and well documented. Overall, the experimental data support the conclusions, which are presented in a balanced and cautious manner. However, several aspects should be improved before the manuscript can be considered for publication in PLOS ONE. In particular, the discussion of the underlying physical mechanisms should be strengthened, certain interpretations related to the bandgap and defect chemistry require further clarification, and the results should be better discussed in the context of recent literature. After addressing the following comments, the manuscript would meet the publication standards of the journal.

Major Comments

1. The authors should provide a more detailed explanation of why Ni exhibits a different behavior in the PMN-PT system compared with alkaline niobates. The discussion should clarify the possible physical and chemical mechanisms responsible for this discrepancy and relate the observations to previous studies.

**Response**: We believe the difference is mainly in the modification and interruption of the energy landscape in the Nb-O framework. We have added a new section dedicated to explaining the role of Ni in PMN-PT and its comparison with the situation in alkaline niobates. Please see the highlight changes under section ‘‘Different roles of Ni^2+^-oxygen vacancy defect dipoles in complex PMN-PT and in simple alkaline niobates’’ in the revised manuscript.

2. The oxidation state of Ni should ideally be confirmed by X-ray Photoelectron Spectroscopy (XPS). If such measurements are not available, the authors should clearly acknowledge this limitation and discuss how it may affect the interpretation of the results.

**Response**: We have added XPS data. Ni is confirmed to be Ni^2+^. Increased concentration of oxygen vacancies is also identified in the Ni^2+^-replacing-Nb^5+^ sample from the XPS data, which significantly assisted explaining the underlying mechanism. We hence substantially revised many relevant parts of the manuscript. Please see the revised Figure 1 as well as various highlighted changes under sections ‘‘Characterization’’, ‘‘Phase, microstructure, and compositions of perovskites’’, and ‘‘Optical and optoelectrical behavior of perovskites’’ in the revised manuscript.

3. The discussion regarding the relationship between the microstructure and the electrical properties should be significantly improved. A deeper analysis linking the observed microstructural features to the dielectric, ferroelectric, piezoelectric, and optoelectrical behaviors would considerably strengthen the manuscript.

**Response**: We have rewritten a large part of the microstructure-property relationship and tried to clarify the influence of both composition and microstructure on various electrical properties. Together with our responses to comments 1 and 2 above, we believe now the analysis is decent. Please see the highlighted changes under sections ‘‘Phases, microstructure, and compositions of perovskites’’, ‘‘Optical and optoelectrical behavior of perovskites’’, and ‘‘Relationship between composition/microstructure and ferroelectric/piezoelectric properties’’ in the revised manuscript.

4. The Introduction is relatively lengthy, and the Conclusions largely repeat results that have already been discussed. The Introduction should be condensed to improve readability, while the Conclusions should focus on the main findings and their scientific significance rather than restating experimental observations.

**Response**: We have rewritten the introduction and conclusions accordingly. The introduction has been significantly condensed, and the conclusions now reflect the main findings and the scientific significance. Please see the highlighted changes under sections ‘‘Introduction’’ and ‘‘Conclusions’’ in the revised manuscript.

Minor Comment

The manuscript would benefit from the inclusion of several recent and relevant references published between 2024 and 2026, particularly in the fields of photoferroelectric materials, bandgap engineering, and optoelectronic ferroelectrics.

**Response**: During revision, we added extra relevant references, including some published between 2024 and 2026, to support our discussions. Examples include references [12], [13], [14], [16], and [30].

Sincerely,

Yang Bai, on behalf of all co-authors
