# Supplementary material for: Effect of Sm and Ni co-doping on optical and electrical properties of lead magnesate niobate titanate ceramics
Source: PLoS One. 2026 Sep 1;21(9):e0357271. doi: 10.1371/journal.pone.0357271 (PMC13533389; doi:10.1371/journal.pone.0357271)
Supplement: Peer review history

# Peer review: Decision Letter 0

**DOI:** 10.1371/journal.pone.0357271.r001

**Authors:** Talaat Abdel Hamid

2 Jul 2026

Dear Dr. Bai,

Thank you for submitting your manuscript to PLOS ONE. After careful consideration, we feel that it has merit but does not fully meet PLOS ONE’s publication criteria as it currently stands. Therefore, we invite you to submit a revised version of the manuscript that addresses the points raised during the review process.

Please submit your revised manuscript by Aug 31 2026 11:59PM. If you will need more time than this to complete your revisions, please reply to this message or contact the journal office at plosone@plos.org. When you're ready to submit your revision, log on to https://www.editorialmanager.com/pone/ and select the 'Submissions Needing Revision' folder to locate your manuscript file.

- A letter that responds to each point raised by the academic editor and reviewer(s). You should upload this letter as a separate file labeled 'Response to Reviewers'.
- A marked-up copy of your manuscript that highlights changes made to the original version. You should upload this as a separate file labeled 'Revised Manuscript with Track Changes'.
- An unmarked version of your revised paper without tracked changes. You should upload this as a separate file labeled 'Manuscript'.

If you would like to make changes to your financial disclosure, please include your updated statement in your cover letter. Guidelines for resubmitting your figure files are available below the reviewer comments at the end of this letter.

If applicable, we recommend that you deposit your laboratory protocols in protocols.io to enhance the reproducibility of your results. Protocols.io assigns your protocol its own identifier (DOI) so that it can be cited independently in the future. For instructions see: https://journals.plos.org/plosone/s/submission-guidelines#loc-laboratory-protocols. Additionally, PLOS ONE offers an option for publishing peer-reviewed Lab Protocol articles, which describe protocols hosted on protocols.io. Read more information on sharing protocols at https://plos.org/protocols?utm_medium=editorial-email&utm_source=authorletters&utm_campaign=protocols.

As the corresponding author, your ORCID iD is verified in the submission system and will appear in the published article. PLOS supports the use of ORCID, and we encourage all coauthors to register for an ORCID iD and use it as well. Please encourage your coauthors to verify their ORCID iD within the submission system before final acceptance, as unverified ORCID iDs will not appear in the published article. *Only*  the individual author can complete the verification step; PLOS staff *cannot*  verify ORCID iDs on behalf of authors.

We look forward to receiving your revised manuscript.

Kind regards,

Talaat Abdel Hamid, Ph.D

Academic Editor

PLOS One

Journal Requirements:

When submitting your revision, we need you to address these additional requirements.

1.Please ensure that your manuscript meets PLOS ONE's style requirements, including those for file naming. The PLOS ONE style templates can be found at

https://journals.plos.org/plosone/s/file?id=wjVg/PLOSOne_formatting_sample_main_body.pdf and

https://journals.plos.org/plosone/s/file?id=ba62/PLOSOne_formatting_sample_title_authors_affiliations.pdf

2. Please note that funding information should not appear in any section or other areas of your manuscript. We will only publish funding information present in the Funding Statement section of the online submission form. Please remove any funding-related text from the manuscript.

3. Thank you for stating the following financial disclosure:

“(1) Funded by the European Union (ERC, UNIFY, 101039110). Views and opinions expressed are however those of the authors only and do not necessarily reflect those of the European Union or the European Research Council Executive Agency. Neither the European Union nor the granting authority can be held responsible for them; (2) Co-funded by the European Research Council (ERC) under the European Union's Horizon 2020 research and innovation programme (grant agreement No. 101002219); (3) Co-funded by the Israel Science Foundation, grant number 1479/21.”

Please state what role the funders took in the study.  If the funders had no role, please state: "The funders had no role in study design, data collection and analysis, decision to publish, or preparation of the manuscript."

If this statement is not correct you must amend it as needed.

Please include this amended Role of Funder statement in your cover letter; we will change the online submission form on your behalf.

4. Please include captions for your Supporting Information files at the end of your manuscript, and update any in-text citations to match accordingly. Please see our Supporting Information guidelines for more information: http://journals.plos.org/plosone/s/supporting-information.

5. If the reviewer comments include a recommendation to cite specific previously published works, please review and evaluate these publications to determine whether they are relevant and should be cited. There is no requirement to cite these works unless the editor has indicated otherwise.

[Note: HTML markup is below. Please do not edit.]

Reviewers' comments:

Reviewer's Responses to Questions

**Comments to the Author**

1. Is the manuscript technically sound, and do the data support the conclusions?

Reviewer #1: Yes

**********

2. Has the statistical analysis been performed appropriately and rigorously?

Reviewer #1: Yes

**********

3. Have the authors made all data underlying the findings in their manuscript fully available?

The PLOS Data policy

Reviewer #1: Yes

**********

4. Is the manuscript presented in an intelligible fashion and written in standard English?

Reviewer #1: Yes

**********

Reviewer #1: Recommendation: Major Revision

The manuscript presents a comprehensive experimental study that is methodologically rigorous and well documented. Overall, the experimental data support the conclusions, which are presented in a balanced and cautious manner. However, several aspects should be improved before the manuscript can be considered for publication in PLOS ONE. In particular, the discussion of the underlying physical mechanisms should be strengthened, certain interpretations related to the bandgap and defect chemistry require further clarification, and the results should be better discussed in the context of recent literature. After addressing the following comments, the manuscript would meet the publication standards of the journal.

Major Comments

1) The authors should provide a more detailed explanation of why Ni exhibits a different behavior in the PMN-PT system compared with alkaline niobates. The discussion should clarify the possible physical and chemical mechanisms responsible for this discrepancy and relate the observations to previous studies.

2) The oxidation state of Ni should ideally be confirmed by X-ray Photoelectron Spectroscopy (XPS). If such measurements are not available, the authors should clearly acknowledge this limitation and discuss how it may affect the interpretation of the results.

3) The discussion regarding the relationship between the microstructure and the electrical properties should be significantly improved. A deeper analysis linking the observed microstructural features to the dielectric, ferroelectric, piezoelectric, and optoelectrical behaviors would considerably strengthen the manuscript.

4) The Introduction is relatively lengthy, and the Conclusions largely repeat results that have already been discussed. The Introduction should be condensed to improve readability, while the Conclusions should focus on the main findings and their scientific significance rather than restating experimental observations.

Minor Comment

The manuscript would benefit from the inclusion of several recent and relevant references published between 2024 and 2026, particularly in the fields of photoferroelectric materials, bandgap engineering, and optoelectronic ferroelectrics.

**********

what does this mean?). If published, this will include your full peer review and any attached files.

If you choose “no”, your identity will remain anonymous but your review may still be made public.

**Do you want your identity to be public for this peer review?**  For information about this choice, including consent withdrawal, please see our Privacy Policy

Reviewer #1: No

**********

[NOTE: If reviewer comments were submitted as an attachment file, they will be attached to this email and accessible via the submission site. Please log into your account, locate the manuscript record, and check for the action link "View Attachments". If this link does not appear, there are no attachment files.]

To ensure your figures meet our technical requirements, please review our figure guidelines: https://journals.plos.org/plosone/s/figures

You may also use PLOS’s free figure tool, NAAS, to help you prepare publication quality figures: https://journals.plos.org/plosone/s/figures#loc-tools-for-figure-preparation.

NAAS will assess whether your figures meet our technical requirements by comparing each figure against our figure specifications.

Attachment Submitted filename: Recommendation Article Plus One.docx

# Author comment: Author response to Decision Letter 1

**DOI:** 10.1371/journal.pone.0357271.r002

28 Jul 2026

Dear Dr. Talaat Abdel Hamid,

Thank very much for handling our manuscript for peer review. We have improved our manuscript based on your and the reviewer’s comments. Please find our response to each of the comments as follows.

Journal Requirements:

When submitting your revision, we need you to address these additional requirements.

1.Please ensure that your manuscript meets PLOS ONE's style requirements, including those for file naming. The PLOS ONE style templates can be found at

https://journals.plos.org/plosone/s/file?id=wjVg/PLOSOne_formatting_sample_main_body.pdf and

https://journals.plos.org/plosone/s/file?id=ba62/PLOSOne_formatting_sample_title_authors_affiliations.pdf

Response: We have followed the instructions provided in the above webpages. We believe our manuscript now meets the PLOS ONE’s style requirements. Please see the highlighted changes throughout the revised manuscript.

2. Please note that funding information should not appear in any section or other areas of your manuscript. We will only publish funding information present in the Funding Statement section of the online submission form. Please remove any funding-related text from the manuscript.

Response: We have removed all funding-related texts from the manuscript.

3. Thank you for stating the following financial disclosure:

“(1) Funded by the European Union (ERC, UNIFY, 101039110). Views and opinions expressed are however those of the authors only and do not necessarily reflect those of the European Union or the European Research Council Executive Agency. Neither the European Union nor the granting authority can be held responsible for them; (2) Co-funded by the European Research Council (ERC) under the European Union's Horizon 2020 research and innovation programme (grant agreement No. 101002219); (3) Co-funded by the Israel Science Foundation, grant number 1479/21.”

Please state what role the funders took in the study. If the funders had no role, please state: "The funders had no role in study design, data collection and analysis, decision to publish, or preparation of the manuscript."

If this statement is not correct you must amend it as needed.

Please include this amended Role of Funder statement in your cover letter; we will change the online submission form on your behalf.

Response: First, we have removed all funding-related texts from the manuscript and will only state the funding in the online submission form. Also, we have stated "The funders had no role in study design, data collection and analysis, decision to publish, or preparation of the manuscript." in the cover letter as well as in the online submission form.

4. Please include captions for your Supporting Information files at the end of your manuscript, and update any in-text citations to match accordingly. Please see our Supporting Information guidelines for more information: http://journals.plos.org/plosone/s/supporting-information.

Response: We have followed the guidelines provided in the above webpage and provided captions for the Supporting Information files at the end of the revised manuscript; please see the highlighted changes.

5. If the reviewer comments include a recommendation to cite specific previously published works, please review and evaluate these publications to determine whether they are relevant and should be cited. There is no requirement to cite these works unless the editor has indicated otherwise.

Response: The reviewer suggested to include extra references but did not specify. We agree with the reviewer and hence have included more related references.

Reviewer #1:

The manuscript presents a comprehensive experimental study that is methodologically rigorous and well documented. Overall, the experimental data support the conclusions, which are presented in a balanced and cautious manner. However, several aspects should be improved before the manuscript can be considered for publication in PLOS ONE. In particular, the discussion of the underlying physical mechanisms should be strengthened, certain interpretations related to the bandgap and defect chemistry require further clarification, and the results should be better discussed in the context of recent literature. After addressing the following comments, the manuscript would meet the publication standards of the journal.

Major Comments

1. The authors should provide a more detailed explanation of why Ni exhibits a different behavior in the PMN-PT system compared with alkaline niobates. The discussion should clarify the possible physical and chemical mechanisms responsible for this discrepancy and relate the observations to previous studies.

Response: We believe the difference is mainly in the modification and interruption of the energy landscape in the Nb-O framework. We have added a new section dedicated to explaining the role of Ni in PMN-PT and its comparison with the situation in alkaline niobates. Please see the highlight changes under section ‘‘Different roles of Ni2+-oxygen vacancy defect dipoles in complex PMN-PT and in simple alkaline niobates’’ in the revised manuscript.

2. The oxidation state of Ni should ideally be confirmed by X-ray Photoelectron Spectroscopy (XPS). If such measurements are not available, the authors should clearly acknowledge this limitation and discuss how it may affect the interpretation of the results.

Response: We have added XPS data. Ni is confirmed to be Ni2+. Increased concentration of oxygen vacancies is also identified in the Ni2+-replacing-Nb5+ sample from the XPS data, which significantly assisted explaining the underlying mechanism. We hence substantially revised many relevant parts of the manuscript. Please see the revised Figure 1 as well as various highlighted changes under sections ‘‘Characterization’’, ‘‘Phase, microstructure, and compositions of perovskites’’, and ‘‘Optical and optoelectrical behavior of perovskites’’ in the revised manuscript.

3. The discussion regarding the relationship between the microstructure and the electrical properties should be significantly improved. A deeper analysis linking the observed microstructural features to the dielectric, ferroelectric, piezoelectric, and optoelectrical behaviors would considerably strengthen the manuscript.

Response: We have rewritten a large part of the microstructure-property relationship and tried to clarify the influence of both composition and microstructure on various electrical properties. Together with our responses to comments 1 and 2 above, we believe now the analysis is decent. Please see the highlighted changes under sections ‘‘Phases, microstructure, and compositions of perovskites’’, ‘‘Optical and optoelectrical behavior of perovskites’’, and ‘‘Relationship between composition/microstructure and ferroelectric/piezoelectric properties’’ in the revised manuscript.

4. The Introduction is relatively lengthy, and the Conclusions largely repeat results that have already been discussed. The Introduction should be condensed to improve readability, while the Conclusions should focus on the main findings and their scientific significance rather than restating experimental observations.

Response: We have rewritten the introduction and conclusions accordingly. The introduction has been significantly condensed, and the conclusions now reflect the main findings and the scientific significance. Please see the highlighted changes under sections ‘‘Introduction’’ and ‘‘Conclusions’’ in the revised manuscript.

Minor Comment

The manuscript would benefit from the inclusion of several recent and relevant references published between 2024 and 2026, particularly in the fields of photoferroelectric materials, bandgap engineering, and optoelectronic ferroelectrics.

Response: During revision, we added extra relevant references, including some published between 2024 and 2026, to support our discussions. Examples include references [12], [13], [14], [16], and [30].

Sincerely,

Yang Bai, on behalf of all co-authors

Attachment Submitted filename: Response letter to reviewers.docx

# Peer review: Decision Letter 1

**DOI:** 10.1371/journal.pone.0357271.r003

**Authors:** Talaat Abdel Hamid

16 Aug 2026

Effect of Sm and Ni co-doping on optical and electrical properties of lead magnesate niobate titanate ceramics

PONE-D-26-26986R1

Dear Dr. Bai,

We’re pleased to inform you that your manuscript has been judged scientifically suitable for publication and will be formally accepted for publication once it meets all outstanding technical requirements.

Within one week, you’ll receive an e-mail detailing the required amendments. When these have been addressed, you’ll receive a formal acceptance letter and your manuscript will be scheduled for publication.

An invoice will be generated when your article is formally accepted. Please note, if your institution has a publishing partnership with PLOS and your article meets the relevant criteria, all or part of your publication costs will be covered. Please make sure your user information is up-to-date by logging into Editorial Manager at Editorial Manager® and clicking the ‘Update My Information' link at the top of the page. For questions related to billing, please contact billing support.

If your institution or institutions have a press office, please notify them about your upcoming paper to help maximize its impact. If they’ll be preparing press materials, please inform our press team as soon as possible -- no later than 48 hours after receiving the formal acceptance. Your manuscript will remain under strict press embargo until 2 pm Eastern Time on the date of publication. For more information, please contact onepress@plos.org.

Kind regards,

Talaat Abdel Hamid, Ph.D

Academic Editor

PLOS One

Additional Editor Comments (optional):

Reviewers' comments:

Reviewer's Responses to Questions

**Comments to the Author**

Reviewer #1: All comments have been addressed

**********

2. Is the manuscript technically sound, and do the data support the conclusions?

Reviewer #1: Yes

**********

3. Has the statistical analysis been performed appropriately and rigorously?

Reviewer #1: Yes

**********

4. Have the authors made all data underlying the findings in their manuscript fully available?

The PLOS Data policy

Reviewer #1: Yes

**********

5. Is the manuscript presented in an intelligible fashion and written in standard English?

Reviewer #1: Yes

**********

Reviewer #1: The authors have satisfactorily addressed all my comments and questions. The article is now ready for publication.

**********

what does this mean?). If published, this will include your full peer review and any attached files.

If you choose “no”, your identity will remain anonymous but your review may still be made public.

**Do you want your identity to be public for this peer review?**  For information about this choice, including consent withdrawal, please see our Privacy Policy

Reviewer #1: No

**********

# Editor report: Acceptance letter

**DOI:** 10.1371/journal.pone.0357271.r004

**Authors:** Talaat Abdel Hamid

PONE-D-26-26986R1

PLOS One

Dear Dr. Bai,

I'm pleased to inform you that your manuscript has been deemed suitable for publication in PLOS One. Congratulations! Your manuscript is now being handed over to our production team.

At this stage, our production department will prepare your paper for publication. This includes ensuring the following:

* All references, tables, and figures are properly cited

* All relevant supporting information is included in the manuscript submission,

* There are no issues that prevent the paper from being properly typeset

You will receive further instructions from the production team, including instructions on how to review your proof when it is ready. Please keep in mind that we are working through a large volume of accepted articles, so please give us a few days to review your paper and let you know the next and final steps.

Lastly, if your institution or institutions have a press office, please let them know about your upcoming paper now to help maximize its impact. If they'll be preparing press materials, please inform our press team within the next 48 hours. Your manuscript will remain under strict press embargo until 2 pm Eastern Time on the date of publication. For more information, please contact onepress@plos.org.

You will receive an invoice from PLOS for your publication fee after your manuscript has reached the completed accept phase. If you receive an email requesting payment before acceptance or for any other service, this may be a phishing scheme. Learn how to identify phishing emails and protect your accounts at https://explore.plos.org/phishing.

If we can help with anything else, please email us at customercare@plos.org.

Thank you for submitting your work to PLOS One and supporting open access.

Kind regards,

PLOS ONE Editorial Office Staff

on behalf of

Dr. Talaat Abdel Hamid

Academic Editor

PLOS One
